# Mathematical Global Dynamics and Control Strategies on *Echinococcus multilocularis* Infection

**DOI:** 10.1155/2019/3569528

**Published:** 2019-06-11

**Authors:** A. S. Hassan, J. M. W. Munganga

**Affiliations:** Department of Mathematical Sciences, College of Science, Engineering and Technology, Florida Science Campus, University of South Africa (UNISA), Pretoria 0003, South Africa

## Abstract

*Echinococcus multilocularis*, a major cause of echinococcosis in human, is a parasitic sylvatic disease between two major hosts in a predator-prey relation. A new model for the transmission dynamics of *Echinococcus multilocularis* in the population of red foxes and voles with environment as a source of infection is formulated and rigorously analyzed. The model is used to access the impact of treatment on red foxes and environmental disinfection as control strategies on the disease dynamics. The control reproduction number is computed and is used to rigorously prove the local and global dynamics of models' equilibria. Using available data on *Echinococcus*, elasticity indices and partial rank correlation coefficients of control reproduction number and cumulative new cases in red foxes and voles are computed. Parameters that have high influence locally and globally are identified. Numerical experiments indicate that administering disinfection of environment only induces more positive impact than applying treatment only on red foxes in controlling the infection. Generally, interventions towards treating red foxes and environmental disinfection could be sufficient in tackling transmission of disease in the populations.

## 1. Introduction

*Echinococcus multilocularis* (EM) is a parasitic taeniid tapeworm and one of the six species of the *Echinococcus* genus. The disease is sylvatic and has indirect life cycle between two major hosts in a predator-prey interaction [1–3]. Adult EM inhabits the small intestine of canines (such as red foxes) which are regarded as definitive hosts and produces eggs that are released to the environment, typically through faeces. After oral ingestion of eggs by rodents (such as voles), regarded as intermediate hosts, a larval stage (metacestode) develops in any of the internal organs (liver, kidney, heart, etc). The mature metacestodes are capable of producing numerous protoscoleces, each having the potential to develop into an adult EM when a definitive host preyed on an intermediate host, and the cycle continues [1, 2, 4, 5]. In some parts of the world, other wild canids (such as coyotes, raccoon dogs, and wolves) can also serve as definitive hosts while other animals (like sheep, domestic dogs, cats, and rats) can be regarded as intermediate hosts [1, 4, 6]. The parasite (EM) causes alveolar echinococcosis in humans regarded as accidental hosts and is characterized with a tumour-like, destructive growth with the potential of causing high fatality rate [1, 4, 7]. The disease was initially confined to a certain part of the globe; however, as researches indicated, it spread all over the globe especially in rural nomadic communities that are economically less privileged, geographically and/or behaviourally detached to certain extent from healthcare systems [8–12]. Furthermore, the disease has high prevalence in red foxes population (1%–76.7%) [3, 5, 13–16] and low prevalence in rodents (voles) (0.4%–30%) [3, 5, 13, 15]. Due to the prevalence of the disease in intermediate hosts and the difficulties involved in treating definitive hosts in a given community, control can take longer times and in many cases may last indefinitely.

Modelling approaches, and in particular mathematical modelling, can give an insight into the biology and epidemiology of diseases in terms of revealing facts on data gaps, understanding the interaction between organisms, predicting future, control, and quantifying certain unmeasurable quantities such as force of infection, basic reproduction number, etc [2]. Mathematical models of *Echinococcus multilocularis* have been developed in literature to attend to must of the above assertions. For instance, in [5], Ishikawa et al. proposed a mathematical model that described the transmission of *Echinococcus multilocularis* in both the definitive (foxes) and intermediate host (voles) populations in Hokkaido. They quantitatively studied seasonal transition in the prevalence of EM in foxes and the risk of infection with human alveolar echinococcosis. Roberts and Aubert [3] developed a simple mathematical model in an attempt to determine the likely effect of combining treatment for EM infection in red foxes and voles in France with the existing vaccination campaign against rabies. It was shown that if the prevalence of the EM in foxes is low in a particular region, the parasite can be eradicated or controlled. Wang et al. [17] proposed a model for the transmission of echinococcosis in dogs, livestock, and human populations to explore effective control and preventive measures in Xianjing. Basic reproduction number, on which the dynamics of model was completely determined, has been estimated, and sensitivity analysis was carried out based on data relevant to the study area. In [18], a model that took into account, the contribution of domestic and stray dogs on the transmission of the parasite in humans was proposed. The global dynamics revealed that, without disposing the stray dogs, the disease became endemic even if the domestic dogs are controlled.

It is a known fact that environment aids the transmission dynamics of *Echinococcus multilocularis*, as discussed in the above reviews. However, this important component is neglected in the modelling process of the disease. In this paper, we establish a noble mathematical model for the transmission dynamics of *Echinococcus multilocularis* within the population of foxes as definitive hosts and the voles as intermediate hosts with concentration of parasites in the environment as a source of infection for intermediate hosts. Considering the high prevalence of EM in red fox population (1–76.7%) compared to rodent population as reported in previous studies, we incorporate treatment as control strategy on the infected red foxes. As a result of the treatment, we assume that recovered red foxes will acquire long-time immunity and as such will not return to susceptible. Furthermore, disinfection or cleaning of environment to reduce the concentration of the disease is also incorporated as a second control strategy. We carry out rigorous analysis on the computation of the basic control reproduction number, a threshold quantity used, to determine the existence and stability dynamics of equilibria. Furthermore, using data available from literature, we conduct elasticity indices of parameters on the control reproduction number and global sensitivity analysis using partial rank correlation coefficients of control reproduction number and cumulative new infections on the two hosts populations. Numerical simulations are conducted to support analytic results and effects of control strategies on the model. This paper is organized as follows. The model formulation, equations, and flow diagram are presented in Section 2. Basic properties of the model on existence, uniqueness, positivity, and boundedness of solutions are discussed in Section 3. Furthermore, existence and global stabilities with systematic calculation of control reproduction number are presented in Section 4. Numerics which comprise of elasticity index, global sensitivity analysis, numerical simulations, and effects of control strategies are presented in Section 5. Finally, we present concluding remarks in Section 6.

## 2. Model Formulation

The total population of red foxes, which is assumed constant (birth and death rates, *μ*_*f*_, are assumed equal) in the environment at time *t*, denoted by *N*_*f*_^*∗*^(*t*) is divided into susceptible (*S*_*f*_(*t*)), exposed (*E*_*f*_(*t*)), infected (*I*_*f*_(*t*)), and recovered (*R*_*f*_(*t*)) subpopulations so that

(1) $$\begin{array}{c} N_{f}^{*}\left(t\right)=S_{f}\left(t\right)+E_{f}\left(t\right)+I_{f}\left(t\right)+R_{f}\left(t\right). \end{array}$$

The susceptible population is increased by recruitment of foxes by birth or immigration at rate *μ*_*f*_*N*_*f*_^*∗*^ and is decreased when it preyed with searching efficiency *s* on an infected vole containing protoscoleces in hydatid cysts [5] with probability *p* of becoming infectious. The exposed fox population is increasing by the number of susceptibles that preyed on infected voles and is decreasing by progression to infected population and natural deaths, at rates *α*_*f*_ and *μ*_*f*_, respectively. The infected fox population is increased by progression of exposed foxes and decreased as a result of treatment and natural deaths at rates *ξ*_*f*_ and *μ*_*f*_, respectively. The population of recovered is increased by the treated infected foxes and decreased by natural death at rates *ξ*_*f*_ and *μ*_*f*_, respectively. We assume here that treated red foxes have either permanent or long-lasting immunity to the parasite and hence will not return to susceptible population.

Similarly, the total population of voles, also assume constant (birth and death rates, *μ*_*v*_, are assumed equal) in the environment at time *t*, denoted by *N*_*v*_^*∗*^(*t*) is subdivided into susceptible (*S*_*v*_(*t*)), exposed (*E*_*v*_(*t*)), and infected (*I*_*v*_(*t*)) subpopulations so that

(2) $$\begin{array}{c} N_{v}^{*}\left(t\right)=S_{v}\left(t\right)+E_{v}\left(t\right)+I_{v}\left(t\right). \end{array}$$

**Figure 1:**
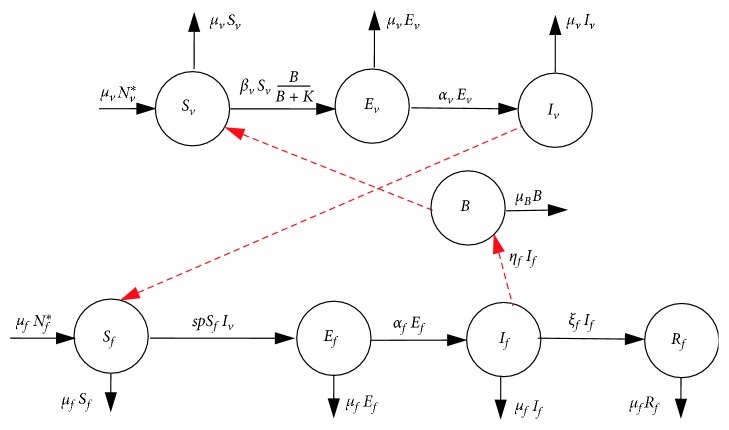
Flow diagram of the transmission dynamics of models (3)–(10).

The population of susceptible voles is increased by birth at rate *μ*_*v*_*N*_*v*_^*∗*^ and decreased by infection from the concentration of parasites in the environment at the rate *β*_*v*_ and natural deaths at rate *μ*_*v*_, which is also applicable to all the subpopulations of voles. Furthermore, the concentration of *Echinococcus* in the environment *B*(*t*) is increased by shedding of the parasites by infected foxes at rate *η*_*f*_ and decreased by disinfection or cleaning of environment at rate *μ*_*b*_. Based on the above descriptions, the model can be described completely by the following system of ordinary differential equations, which follow from the schematic diagram shown in Figure 1:

(3) $$\begin{array}{c} \frac{dS_{f}}{dt}=\mu_{f}N_{f}^{*}-spI_{v}S_{f}-\mu_{f}S_{f}, \end{array}$$

(4) $$\begin{array}{c} \frac{dE_{f}}{dt}=spI_{v}S_{f}-\left(\mu_{f}+\alpha_{f}\right)E_{f}, \end{array}$$

(5) $$\begin{array}{c} \frac{dI_{f}}{dt}=\alpha_{f}E_{f}-\left(\mu_{f}+\xi_{f}\right)I_{f}, \end{array}$$

(6) $$\begin{array}{c} \frac{dR_{f}}{dt}=\xi_{f}I_{f}-\mu_{f}R_{f}, \end{array}$$

(7) $$\begin{array}{c} \frac{dS_{v}}{dt}=\mu_{v}N_{v}^{*}-\beta_{v}\left(\frac{B}{K+B}\right)S_{v}-\mu_{v}S_{v}, \end{array}$$

(8) $$\begin{array}{c} \frac{dE_{v}}{dt}=\beta_{v}\left(\frac{B}{K+B}\right)S_{v}-\left(\mu_{v}+\alpha_{v}\right)E_{v}, \end{array}$$

(9) $$\begin{array}{c} \frac{dI_{v}}{dt}=\alpha_{v}E_{v}-\mu_{v}I_{v}, \end{array}$$

(10) $$\begin{array}{c} \frac{dB}{dt}=\eta_{f}I_{f}-\mu_{b}B, \end{array}$$

with initial conditions

(11) $$\begin{array}{c} S_{f}\left(0\right)>0,E_{f}>0,I_{f}\left(0\right)>0,R_{f}\left(0\right)>0,S_{v}\left(0\right)>0,\\ E_{v}\left(0\right)>0,I_{v}\left(0\right)>0,B\left(0\right)>0. \end{array}$$

## 3. Basic Properties of the Model

The model (3)–(10) monitors the dynamics of red foxes and voles populations, and all its associated parameters are assumed nonnegative. Hence, we now present the basic results for the properties of the model.

**Theorem 1 .**

The following region is positively invariant for the model (3): Ω=Ω_*f*_ × Ω_*v*_ × Ω_*b*_ ⊂ *ℝ*_+_^4^ × *ℝ*_+_^3^ × *ℝ*_+_, where Ω_*f*_={(*S*_*f*_, *E*_*f*_, *I*_*f*_, *R*_*f*_) ⊂ *ℝ*_+_^4^ : *S*_*f*_+*E*_*f*_+*I*_*f*_+*R*_*f*_=*N*_*f*_^*∗*^}, Ω_*v*_={(*S*_*v*_, *E*_*v*_, *I*_*v*_) ⊂ *ℝ*_+_^3^ : *S*_*v*_+*E*_*v*_+*I*_*v*_=*N*_*v*_^*∗*^} and Ω_*b*_={*B* ⊂ *ℝ*_+_ : *B* ≤ (*η*_*f*_*N*_*f*_^*∗*^/*μ*_*b*_)}.

**Proof**

The detailed proof of Theorem 1 is presented in Appendix A.

Equations (A.14)–(A.18) establish the boundedness of total populations for red foxes, voles, and concentration of parasites, respectively, and by extension verifies the boundedness of subpopulations. Thus, region *Ω* is positively invariant. Hence, in this region, the model (3)–(10) is considered to be mathematically and epidemiologically well posed, and therefore, the dynamics of the model can be studied in *Ω*.

## 4. Existence and Stability of Equilibria

### 4.1. Disease-Free Equilibrium (DFE)

The DFE of the model is obtained by equating the right-hand sides of model equations (3)–(10) to zero as follows:

(12) $$\begin{array}{c} {ℰ}_{0}=\left(S_{f}^{0},E_{f}^{0},I_{f}^{0},R_{f}^{0},S_{v}^{0},E_{v}^{0},I_{v}^{0},B^{0}\right)=\left(N_{f}^{*},0,0,0,N_{v}^{*},0,0,0\right). \end{array}$$

### 4.2. Calculation of Control Reproduction Number

The basic reproduction number in epidemic models is an important threshold value that quantifies the infection risk in order to effectively control the disease. Furthermore, it plays a vital role in stability analysis of equilibria of the models. It can be derived using the next-generation matrix approach [19]. However, when there is intervention, it is referred as the control reproduction number. For detailed computation of the control reproduction number, refer Appendix B. Therefore, the basic control reproduction number, denoted by *ℛ*_*c*_, is given by the following equation:

(13) $$\begin{array}{c} {ℛ}_{c}=\rho \left(FV^{-1}\right)={\left[\left(\frac{N_{f}^{*}sp\alpha_{f}}{{\bar{\alpha }}_{f}{\bar{\xi }}_{f}}\right)\left(\frac{N_{v}^{*}\beta_{v}\alpha_{v}}{\mu_{v}{\bar{\alpha }}_{v}}\right)\left(\frac{\eta_{f}}{K\mu_{b}}\right)\right]}^{1/3}. \end{array}$$

It is worth stating that *ℛ*_*c*_ is the basic control reproduction number, which represents the number of secondary infection cases generated by introducing at least one infective agent into the population that is assumed wholly susceptible. This number is obtained from the contribution of average number of secondary infections through fox-to-environment-to-vole transmission $\left({ℛ}_{c}^{f}=N_{f}^{*}sp\alpha_{f}/{\bar{\alpha }}_{f}{\bar{\xi }}_{f}\right)$, voles-to-fox transmission $\left({ℛ}_{c}^{v}=N_{v}^{*}\beta_{v}\alpha_{v}/\mu_{v}{\bar{\alpha }}_{v}\right)$, and environment-to-voles transmission (*ℛ*_*c*_^*b*^=*η*_*f*_/*Kμ*_*b*_) as a result of one infectious subject during its infectious period.

#### 4.2.1. Stability of DFE

**Theorem 2 .**

The disease-free equilibrium is locally asymptotically stable when *ℛ*_*c*_ < 1 and unstable if *ℛ*_*c*_ > 1.

**Proof**

The local asymptotic stability of DFE is established using Theorem 2 in [19].

The epidemiological implication of the result in Theorem 2 is that the *Echinococcus m*. can be eliminated from the populations when *ℛ*_*c*_ < 1 if the initial sizes of subpopulations are within the basin of attraction of the DFE. In order to guarantee the total elimination of the disease irrespective of the initial population started with in *Ω*, it is necessary to prove the global asymptotic stability (in *Ω*) of the DFE, which is shown below. Here, we use the matrix-theoretic method as described in [20].

**Theorem 3 .**

The DFE of the model (3)–(10) given by (12) is globally asymptotically stable (GAS) in Ω when *ℛ*_*c*_ < 1. If *ℛ*_*c*_ > 1, the DFE is unstable, the system is uniformly persistent, and there exists at least one endemic equilibrium in the interior of Ω.

**Proof**

Let *F* and *V* matrices be given as above. The matrix *V*^−1^*F* is computed to be

(14) $$\begin{array}{c} V^{-1}F=\left[\begin{array}{cccccc} 0 & 0 & 0 & 0 & \frac{spN_{f}^{*}}{{\bar{\alpha }}_{f}} & 0\\ 0 & 0 & 0 & 0 & \frac{\alpha_{f}spN_{f}^{*}}{{\bar{\alpha }}_{f}{\bar{\xi }}_{f}} & 0\\ 0 & 0 & 0 & 0 & \frac{\xi_{f}\alpha_{f}spN_{f}^{*}}{{\bar{\alpha }}_{f}{\bar{\xi }}_{f}\mu_{f}} & 0\\ 0 & 0 & 0 & 0 & 0 & \frac{\beta_{v}N_{v}^{*}}{{\bar{\alpha }}_{v}K}\\ 0 & 0 & 0 & 0 & 0 & \frac{\alpha_{v}\beta_{v}N_{v}^{*}}{{\bar{\alpha }}_{v}\mu_{v}K}\\ 0 & \frac{\eta_{f}}{\mu_{b}} & 0 & 0 & 0 & 0 \end{array}\right]. \end{array}$$

Observe that the matrix *V*^−1^*F* is reducible (second, fifth, and sixth columns are the nonzero columns); hence, Theorem 2.2 of [20] is not applicable, and instead we use the conditions of Theorem 2.1 in [20] to construct the Lyapunov function. Define *X*=(*E*_*f*_, *I*_*f*_, *R*_*f*_, *E*_*v*_, *I*_*v*_, *B*)^*T*^ and *Y*=(*S*_*f*_, *S*_*v*_) so that the dynamics of infected compartments can be expressed as follows:

(15) $$\begin{array}{c} \frac{dX}{dt}=\left(F-V\right)X-f\left(X,Y\right), \end{array}$$

where

(16) $$\begin{array}{c} f\left(X,Y\right)=\left[\begin{array}{c} spI_{v}\left(S_{f}^{0}-S_{f}\right)\\ 0\\ 0\\ \frac{\beta_{v}B}{K\left(K+B\right)}\left(S_{v}^{0}K+\left(S_{v}^{0}-S_{v}\right)K\right)\\ 0\\ 0 \end{array}\right]\geq 0, \end{array}$$

since *S*_*f*_^0^ ≥ *S*_*f*_ and *S*_*v*_^0^ ≥ *S*_*v*_ inside *Ω*.

We define the Lyapunov function as *L*=**u**^*T*^*V*^−1^*X*, where **u** is the left eigenvector of the matrix *V*^−1^*F* with respect to the eigenvalue *ℛ*_*c*_. Thus,

(17) $$\begin{array}{c} \left(u_{1},u_{2},u_{3},u_{4},u_{5},u_{6}\right)V^{-1}F={ℛ}_{c}\left(u_{1},u_{2},u_{3},u_{4},u_{5},u_{6}\right). \end{array}$$

Multiplying and equating (17), we have

(18) $$\begin{array}{c} \left(0,\;\frac{u_{6}\eta_{f}}{\mu_{b}},\;0,\;0,\;\frac{u_{1}spN_{f}^{*}}{{\bar{\alpha }}_{f}}+\frac{u_{2}\alpha_{f}spN_{f}^{*}}{{\bar{\alpha }}_{f}{\bar{\xi }}_{f}}+\frac{u_{3}\alpha_{f}spN_{f}^{*}\xi_{f}}{{\bar{\alpha }}_{f}{\bar{\xi }}_{f}\mu_{f}},\;\frac{u_{4}\beta_{v}N_{v}^{*}}{{\bar{\alpha }}_{v}K}+\frac{u_{5}\alpha_{v}\beta_{v}N_{v}^{*}}{{\bar{\alpha }}_{v}\mu_{v}K}\right)={ℛ}_{c}\left(u_{1},\;u_{2},\;u_{3},\;u_{4},\;u_{5},\;u_{6}\right). \end{array}$$

Clearly, it can be seen that *u*_1_=0,  *u*_3_=0, and *u*_4_=0, and we can deduce that

(19) $$\begin{array}{c} \frac{u_{2}\alpha_{f}spN_{f}^{*}}{{\bar{\alpha }}_{f}{\bar{\xi }}_{f}}={ℛ}_{c}u_{5},\\ \frac{u_{5}\alpha_{v}\beta_{v}N_{v}^{*}}{{\bar{\alpha }}_{v}\mu_{v}K}={ℛ}_{c}u_{6},\\ \frac{u_{6}\eta_{f}}{\mu_{b}}={ℛ}_{c}u_{2}. \end{array}$$

If we take *u*_6_=1, one solution of **u** is as follows:

(20) $$\begin{array}{c} \left(u_{1},\;u_{2},\;u_{3},\;u_{4},\;\;u_{5},\;u_{6}\right)=\left(0,\;\frac{u_{5}{ℛ}_{c}{\bar{\alpha }}_{f}{\bar{\xi }}_{f}}{\alpha_{f}spN_{f}^{*}},\;0,\;0,\;\frac{{ℛ}_{c}{\bar{\alpha }}_{v}K\mu_{v}}{\alpha_{v}\beta_{v}N_{v}^{*}},\;1\right). \end{array}$$

The Lyapunov function *L* is now given as

(21) $$\begin{array}{c} L=\left(u_{1},\;u_{2},\;u_{3},\;u_{4},\;u_{5},\;u_{6}\right)V^{-1}{\left(E_{f},\;I_{f},\;R_{f},\;E_{v},\;I_{v},\;B\right)}^{T}\\ =\left(\frac{u_{5}{ℛ}_{c}}{spN_{f}^{*}}E_{f}+\frac{u_{5}{ℛ}_{c}{\bar{\alpha }}_{f}}{\alpha_{f}spN_{f}^{*}}I_{f}+\frac{{ℛ}_{c}K}{\beta_{v}N_{v}^{*}}E_{v}+\frac{{ℛ}_{c}{\bar{\alpha }}_{v}K}{\alpha_{v}\beta_{v}N_{v}^{*}}I_{v}+\frac{1}{\mu_{b}}B\right)\geq 0. \end{array}$$

Differentiating *L* along the solutions of infected compartments in (3)–(10) gives

(22) $$\begin{array}{c} \frac{dL}{dt}=\frac{{ℛ}_{c}KBS_{v}}{\left(K+B\right)N_{v}^{*}}+\frac{u_{5}{ℛ}_{c}I_{v}S_{f}}{N_{f}^{*}}-\frac{u_{5}{ℛ}_{c}{\bar{\alpha }}_{f}{\bar{\xi }}_{f}I_{f}}{\alpha_{f}spN_{f}^{*}}-\frac{{ℛ}_{c}{\bar{\alpha }}_{v}K\mu_{v}I_{v}}{\alpha_{v}\beta_{v}N_{v}^{*}}+\frac{\eta_{f}I_{f}}{\mu_{b}}-B. \end{array}$$

Using (13) and (21), simplifying and rearranging, we have

(23) $$\begin{array}{c} \frac{dL}{dt}=\left({ℛ}_{c}-1\right)\left(\frac{u_{5}{ℛ}_{c}{\bar{\alpha }}_{f}{\bar{\xi }}_{f}I_{f}}{\alpha_{f}spN_{f}^{*}}+\frac{{ℛ}_{c}{\bar{\alpha }}_{v}K\mu_{v}I_{v}}{\alpha_{v}\beta_{v}N_{v}^{*}}+B\right)+{ℛ}_{c}B\left(\frac{K}{K+B}\frac{S_{v}}{N_{v}^{*}}-1\right)+\frac{{ℛ}_{c}^{2}{\bar{\alpha }}_{v}K\mu_{v}I_{v}}{\alpha_{v}\beta_{v}N_{v}^{*}}\left(\frac{S_{f}}{N_{f}^{*}}-1\right). \end{array}$$

From (23), (*K*/(*K*+*B*)) < 1 and (*S*_*v*_/*N*_*v*_^*∗*^) ≤ 1, and then (*K*/(*K*+*B*))(*S*_*v*_/*N*_*v*_^*∗*^) < 1 and (*S*_*f*_/*N*_*f*_^*∗*^) ≤ 1. Therefore, if *ℛ*_*c*_ < 1, it implies that (*dL*/*dt*) ≤ 0. Furthermore, (*dL*/*dt*)=0 implies either *E*_*f*_=0, *I*_*f*_=0, *R*_*f*_=0, *E*_*v*_=0, *I*_*f*_=0, or *B*=0. Thus, the largest invariant set where (*dL*/*dt*)=0 is the singleton (*ℰ*_0_). Therefore, by LaSalle's invariance principle [21], *ℰ*_0_ is GAS in *Ω* if *ℛ*_*c*_ < 1. Furthermore, if *ℛ*_*c*_ > 1 in (23), the first term is positive, while the second and third terms will be zero in *Ω* when *I*_*v*_=*B*=0; therefore, (*dL*/*dt*) > 0, and hence, *ℰ*_0_ is unstable. Using the argument in Theorem 2.2 of Shuai and van den Driessche [20], it can be shown that the instability of *ℰ*_0_ when *ℛ*_*c*_ > 1 implies that the system is uniformly persistent in *Ω*, thus implying the existence of at least one positive endemic equilibrium.

### 4.3. Existence and Global Stability of Endemic Equilibrium

The existence of endemic equilibrium follows from the argument in Theorem 3. In the presence of disease in the community, the endemic equilibrium *ℰ*_1_ is obtained by setting the right-hand sides of equations (3)–(10) to zero, and thus,

(24) $$\begin{array}{c} {ℰ}_{1}=\left(S_{f}^{*},E_{f}^{*},I_{f}^{*},R_{f}^{*},S_{v}^{*},E_{v}^{*},I_{v}^{*},B^{*}\right), \end{array}$$

where

(25) $$\begin{array}{c} S_{f}^{*}=\frac{\mu_{f}N_{f}^{*}}{spI_{v}^{*}+\mu_{f}},\\ E_{f}^{*}=\frac{spI_{v}^{*}S_{f}^{*}}{{\bar{\alpha }}_{f}},\\ I_{f}^{*}=\frac{\alpha_{f}E_{f}^{*}}{{\bar{\xi }}_{f}},\\ R_{f}^{*}=\frac{\xi_{f}I_{f}^{*}}{\mu_{f}},\\ S_{v}^{*}=\frac{\mu_{v}N_{v}^{*}}{\left(\beta_{v}B^{*}/\left(\kappa +B^{*}\right)\right)+\mu_{v}},\\ E_{v}^{*}=\beta_{v}\frac{B^{*}}{\kappa +B^{*}}\frac{S_{v}^{*}}{{\bar{\alpha }}_{v}},\\ I_{v}^{*}=\frac{\alpha_{v}E_{v}^{*}}{\mu_{v}},\\ B^{*}=\frac{\eta_{f}I_{f}^{*}}{\mu_{b}}. \end{array}$$

**Theorem 4 .**

If *ℛ*_*c*_ > 1, then the unique endemic equilibrium *ℰ*_1_ of model (3)–(10) is globally asymptotically stable (GAS) in Ω.

**Proof**

To prove the uniqueness and global stability of *ℰ*_1_, we apply the method of graph-theoretic as described in Section 3 of [20]. Detailed proof of the theorem is also given in Appendix C.

## 5. Numerics: Elasticity Indices, Numerical Simulations, and Control Strategies

In this section, we use the parameter values in Table 1 with the aim of illustrating the theoretical results and quantifying the control measures for *Echinococcus multilocularis*.

### 5.1. Elasticity Indices

As evident from the expression of basic control reproduction number, *ℛ*_*c*_1 in (13), it is interesting to know qualitatively and estimate quantitatively how perturbations of associated parameters have influence on *ℛ*_*c*_. In order to achieve this, we determine the normalized forward sensitivity index as introduced in Chitnis et al. [22], otherwise called elasticity indices [23] of parameters on *ℛ*_*c*_. This quantity *ϒ* for *ℛ*_*c*_ with respect to a parameter *p* is defined as follows:

(26) $$\begin{array}{c} {ϒ}_{p}^{{ℛ}_{c}}=\frac{\partial {ℛ}_{c}}{\partial p}\times \frac{p}{{ℛ}_{c}}. \end{array}$$

In Table 2, we compute the elasticity indices of *ℛ*_*c*_ with respect to the parameters at static baseline values as indicated in Table 1 and arranged in the descending order of magnitudes. The computations indicate equal influence of six parameters associated with incidences for transmission of the parasites (*β*_*v*_, *s*, *p*, *η*_*f*_), disinfection rate (*μ*_*b*_), and concentration of *Echinococcus* in the environment (*K*). Most importantly, treatment of red foxes (*ξ*_*f*_) has the second largest value followed by incubation rates of voles and foxes in that order.

### 5.2. Global Sensitivity Analysis

**Figure 2:**
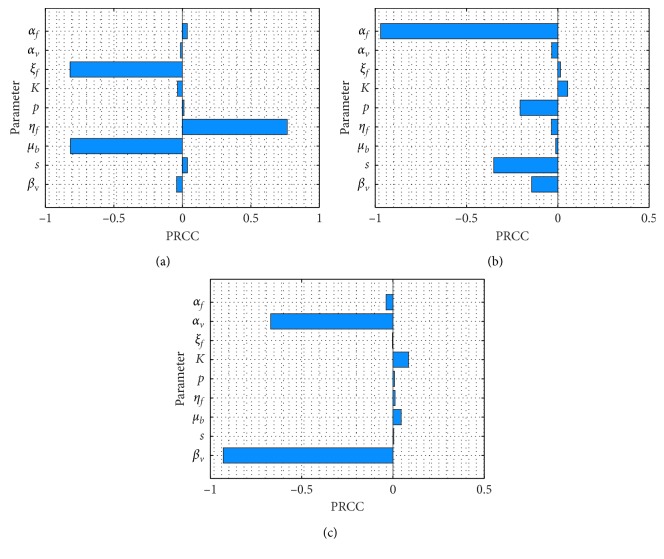
Global sensitivity analysis displaying the partial rank correlation coefficients (PRCC) of (a) control reproduction number *ℛ*_*c*_, (b) cumulative new cases in red foxes, and (c) cumulative new cases in voles populations.

From our result in Section 5.1, it is obvious that the local sensitivity analysis on *ℛ*_*c*_ could not explicitly differentiate the most influential parameters and thus the need for global sensitivity analysis. We adapt the approach in [24] to analyze the global sensitivity of the parameters on both *ℛ*_*c*_ and cumulative new cases in rodents and red foxes, respectively. Using the method of partial rank correlation coefficients (PRCC), as described and implemented in [25], we carry out the global sensitivity analysis of 9 parameters on the control reproduction number *ℛ*_*c*_ and cumulative new infections in the populations of both red foxes and rodents. The main objective is to determine the most influential parameters for the purpose of control and extent of infectivity in the two populations. To compute the PRCC values, we used the MatLab R2017b with ranges of parameters in Table 2 divided into 1000 sample sizes, and the results are displayed in Figure 2. The parameter with the PRCC value far away from zero indicates the more influential parameter is on both *ℛ*_*c*_ and cumulative new cases.

In Figure 2(a), the global sensitivity of parameters on *ℛ*_*c*_ is depicted. It can be seen that the rates of cleaning/disinfecting the environment (*μ*_*b*_) and rate of treating red foxes (*ξ*_*f*_) have the most global influence on *ℛ*_*c*_, followed by rate of red foxes contribution of *E. multilocularis* to the environment (*η*_*f*_). The global sensitivity of parameters on the cumulative number of new cases for red foxes is also displayed in Figure 2(b) which indicates that the incubation rate in red foxes (*α*_*f*_) has the highest global influence, followed by the rate of searching efficiency of red foxes (*s*) and probability that an infected vole preyed on infects a red fox (*p*) in that order. Lastly, in Figure 2(c), the global sensitivity of parameters on cumulative new infection cases in rodents indicates that the transmission rate from environment to rodents (*β*_*v*_) is the most global influential parameter, followed by the incubation rate in rodents (*α*_*v*_). From the global sensitivity analysis, for control purposes, it can be suggested that more emphasis should be given to cleaning/disinfecting the environment, for example, by removing carcass and administering praziquantel to red foxes.

### 5.3. Numerical Simulations

**Figure 3:**
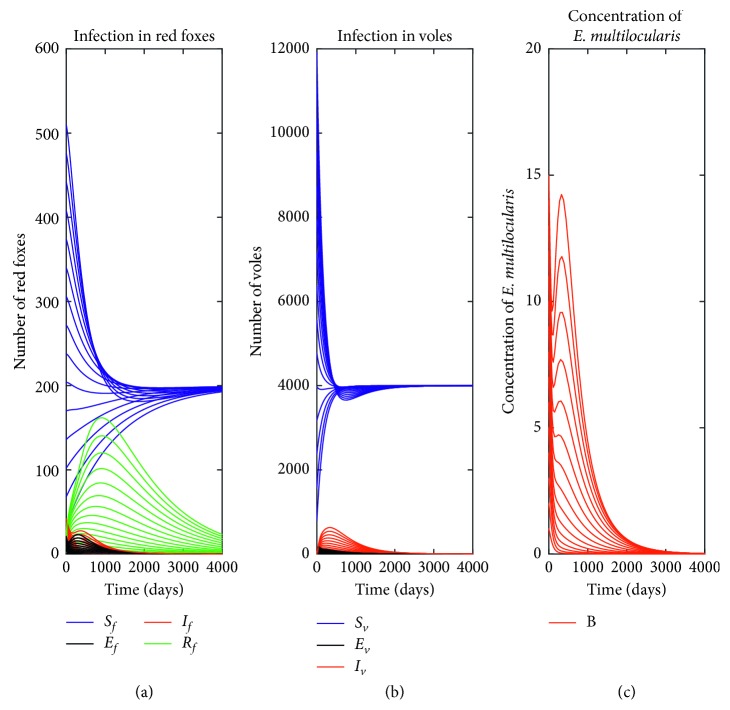
Time evolution of (a) red foxes, (b) voles, and (c) concentration of *E. multilocularis* using different initial conditions when there is no infection with parameter values in Table 1, which gives the control reproduction number *ℛ*_*c*_=0.97.

Figure 3 depicts the global stability of disease-free equilibrium as proved in Theorem 3 with different initial conditions, where the numbers of foxes, voles, and concentration of *E. multilocularis* converges asymptotically to the equilibrium point using different initial conditions. The parameter values in Table 1 are used so that the control reproduction number *ℛ*_*c*_=0.97 < 1. It can be seen that all disease compartments (*E*_*f*_^*∗*^, *I*_*f*_^*∗*^, *R*_*f*_^*∗*^, *E*_*v*_^*∗*^, *I*_*v*_^*∗*^,  *and* *B*^*∗*^) converge asymptotically to zero while the noninfected compartments (*S*_*f*_^*∗*^ *and* *S*_*v*_^*∗*^) converge to their respective total populations.

**Figure 4:**
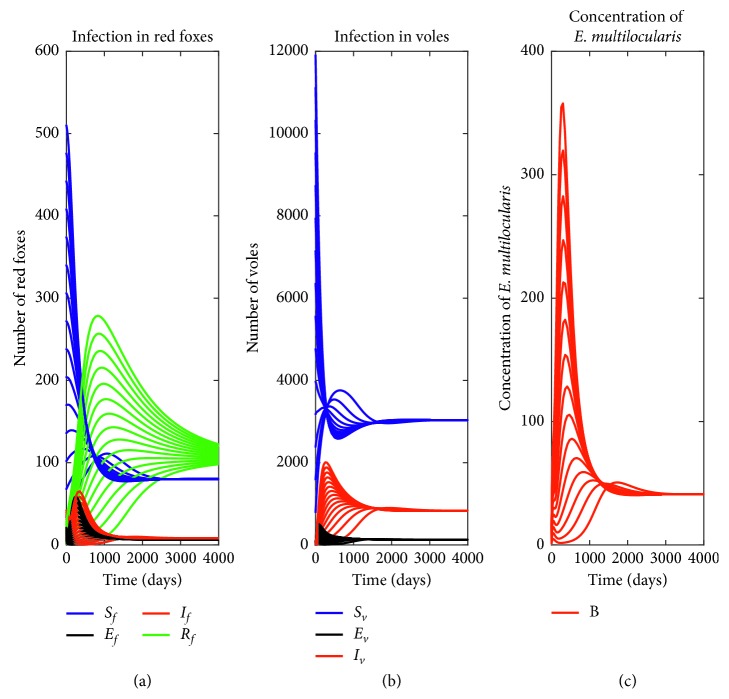
Time evolution of (a) red foxes, (b) voles, and (c) concentration of *E. multilocularis* with different initial conditions when there is infection using parameter values in Table 1, except for *ξ*_*f*_=0.2 with control reproduction number *ℛ*_*c*_=1.94.

In Figure 4, the time evolution for number of red foxes, voles, and the concentration of *Echinococcus multilocularis* for model (3)–(10) is illustrated using the parameter values in Table 1, except for *η*_*f*_=0.2 depicting GAS of endemic equilibrium as proved in Theorem 4. It can be observed that populations of foxes, voles, and concentration of parasite converge asymptotically to their respective endemic equilibrium points irrespective of the initial population started with. Here, the control reproduction number *ℛ*_*c*_=1.94 > 1. Hence, the disease will persist in the community.

### 5.4. Effects of Control Strategies on *ℛ*_*c*_

In this section, the effects of treating red foxes (*ξ*_*f*_) and the cleaning or disinfecting the environment (*μ*_*b*_) are going to be explored. So here it will be interesting to see how values of infectious red foxes and/or the *ℛ*_*c*_ will change as these parameters are varied when other parameters are fixed at the baseline values.

**Figure 5:**
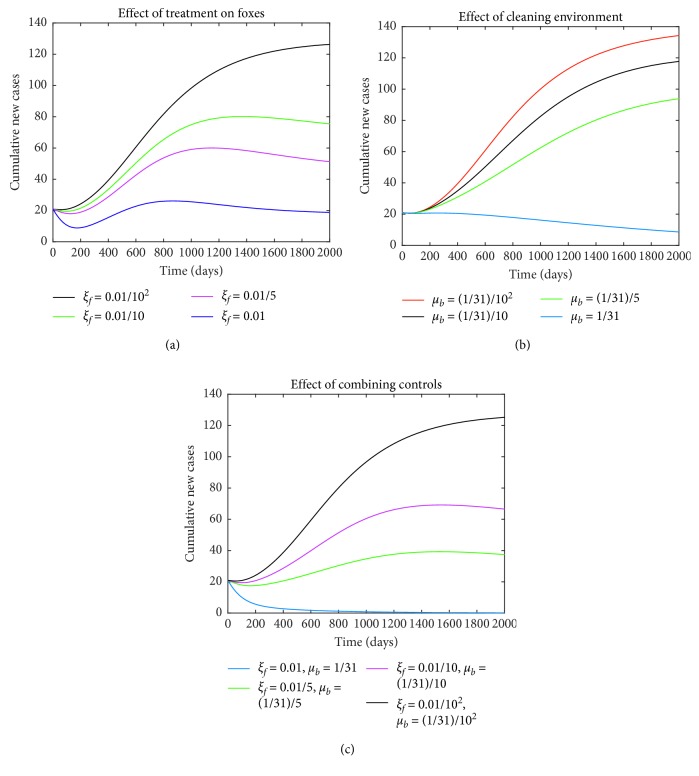
Numerical simulations displaying effects of control strategies on the cumulative number of infectious red foxes, using parameter values in Table 1 with varying values of *μ*_*b*_ and *ξ*_*f*_, except in (a) effect of treating red foxes only (*μ*_*b*_=0), (b) effect of environmental cleaning only (*ξ*_*f*_=0), and (c) effect of combining controls.

The effect of treatment-only on red foxes is illustrated in Figure 5(a) using the baseline parameter values in Table 1 except for *μ*_*b*_=0. The value of *ξ*_*f*_ is varied from *ξ*_*f*_=0.01/10^2^ to *ξ*_*f*_=0.01; as a result, the number of infectious red foxes is reduced from 126 to 19, respectively, as displayed by the graphs. Similarly, the effect of controlling the red foxes by cleaning and/or disinfecting environment-only is shown in Figure 5(b) with fixed parameter values except *ξ*_*f*_=0, and the value of *μ*_*b*_ varied from *μ*_*b*_=(1/31)/10^2^ to *μ*_*b*_=(1/31). It can be seen that the cumulative number of infectious red foxes decrease from 134 with *ℛ*_*c*_=10.01 to 8, *ℛ*_*c*_=2.16, respectively. It is evident that the implementation of either of the two control strategies may not be adequate in eradicating the parasite completely from the community. Therefore, when the control strategies are administered simultaneously, as depicted in Figure 5(c), the cumulative number of infectious red foxes decreases from 125 with *ℛ*_*c*_=9.60 to 0, with *ℛ*_*c*_=0.80, respectively. Hence, combining the two control strategies is more effectively followed by environmental cleaning/disinfection and treatment of red foxes. The later results agree with our elasticity indices in Section 5.1 for the two parameters (*ξ*_*f*_ and *μ*_*b*_).

**Figure 6:**
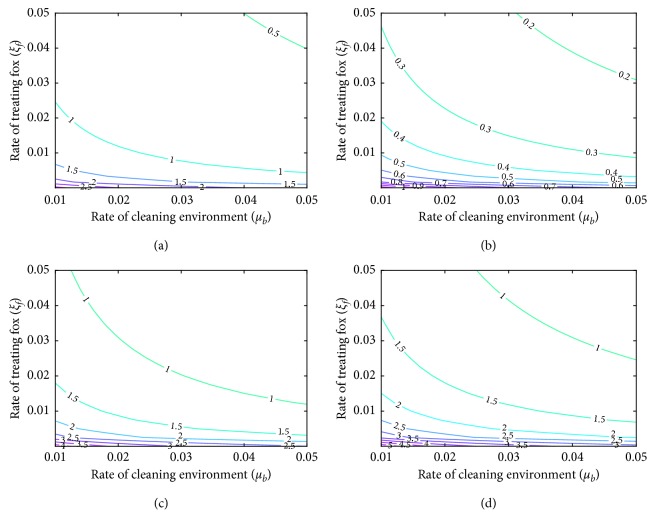
Contour curves of *ℛ*_*c*_ as a function of *ξ*_*f*_ and *μ*_*b*_ using three different rates of red foxes contribution to environment (*η*_*f*_), with (a) parameter values in Table 1, in (b) *η*_*f*_=0.001, (c) *η*_*f*_=0.10, and (d) *η*_*f*_=0.20.

Given that *ξ*_*f*_ and *μ*_*b*_ are the control parameters in the model, it is important to see how *ℛ*_*c*_ varies as the two parameters are varied with others fixed using contour plots. The objective is to estimate values of *ξ*_*f*_ and *μ*_*b*_ that will ensure disease eradication (making *ℛ*_*c*_ < 1) as stated in Theorem 2. The results are displayed as contour curves of *ℛ*_*c*_ as a function of treatment on red foxes (*ξ*_*f*_) and cleaning/disinfection of environment (*μ*_*b*_) at fixed baseline values in Figure 6(a). The least values of *ξ*_*f*_ and *μ*_*b*_ that will ensure parasites eradication are estimated to be 0.025 and 0.01 so that *ℛ*_*c*_=0.99 or 0.005 and 0.05 with *ℛ*_*c*_=0.95, respectively. Furthermore, to access the impact of combined treatment and reduced contribution of parasite to environment by red foxes, contour plots of *ℛ*_*c*_ as function of the control strategies with varying rate of contribution by red foxes to the environment (*η*_*f*_) are displayed in Figures 6(b)–6(d). The figures show remarkable increase in the associated control reproduction number with increase in rate of contribution of parasites to environment by red foxes. In Figure 6(b), low control strategies are needed if the rate of contribution (*η*_*f*_=0.001) is very small to ensure almost total eradication of the parasites, with range of *ℛ*_*c*_ ∈ [0.17, 1.17] and *mean* = 0.67. In Figure 6(c), with high contribution rate (*η*_*f*_=0.2), the control strategies must also be high to lower the value of *ℛ*_*c*_ ∈ [0.99, 6.84] with *mean* = 3.92. However, when the rate of contribution (*η*_*f*_=0.1) is moderate, in Figure 6(b), the control strategies must be in reciprocal combinations (low treatment rate versus high disinfection rate and vice versa) putting the range of *ℛ*_*c*_ ∈ [0.79, 5.43] with *mean* = 3.11.

## 6. Concluding Remarks

A new global deterministic model for the transmission of *Echinococcus multilocularis* in the population of red foxes and voles with environment as a source of infection is formulated and used to access the impact of control strategies on the disease dynamics. Moreover, sensitivity analysis is carried out to determine the parameters that have influence on the control reproduction number and cumulative new infectious cases of red foxes and rodents. We start by investigating the basic properties of the model to ascertain its worthiness mathematically and epidemiologically. The major findings of the study are outlined as follows:

1. The disease-free equilibrium of the model is obtained and used to systematically determine the basic control reproduction number (*ℛ*_*c*_). Furthermore, using a matrix-theoretic method, the DFE is globally asymptotically stable whenever *ℛ*_*c*_ is less than unity. The implication of this result is that infection of the parasite can be control in the community if *ℛ*_*c*_ can be reduced and maintain below unity.
2. When the control reproduction number exceeds unity, a unique endemic equilibrium exists, and using a graph-theoretic method, it is shown to be globally asymptotically stable if *ℛ*_*c*_ is greater than unity. Hence, the infection will persist in the community when the control strategies fail to reduce *ℛ*_*c*_ below unity.
3. Elasticity indices of *ℛ*_*c*_ on key parameters are computed, and the results indicate equal influence of six parameters associated with incidence for transmission of the disease (*β*_*v*_, *s*, *p*,  *and* *η*_*f*_), cleaning/disinfection (*μ*_*b*_), and concentration of parasite in the environment (*K*), followed by rate of treatment on foxes (*ξ*_*f*_) and incubation rates of voles (*α*_*v*_) and foxes (*α*_*f*_) in that order.
4. Having noticed that the local sensitivity analysis on *ℛ*_*c*_ could not differentiate explicitly the most influential parameter(s) of the model, a global sensitivity using PRCC is conducted. From the simulations, the two control parameters: rate of cleaning/disinfecting the environment (*μ*_*b*_) and rate of treating red foxes have the most global influence on *ℛ*_*c*_, followed by rate of red foxes contribution of *E. multilocularis* to the environment (*η*_*f*_). The global sensitivity of parameters on the cumulative number of new cases for red foxes indicates that the incubation rate in red foxes (*α*_*f*_) has the highest global influence, followed by rate of searching efficiency of red foxes (*s*) and probability that an infected vole preyed on infects a red fox (*p*) in that order. On the contrary, the global sensitivity of parameters on cumulative new infection cases in rodents shows that the transmission rate from environment to rodents (*β*_*v*_) is the most global influential parameter, followed by incubation rate in rodents (*α*_*v*_).
5. Numerical simulations with baseline values and varying *ξ*_*f*_ and *μ*_*b*_, have indicated that administering disinfection of environment only induce more positive control impact (leaving only 8 infected red foxes) compared to applying treatment only on red foxes (leaving about 19 infected red foxes). However, administering the two control strategies induce the most positive impact by treating all the infected red foxes.
6. Contour curves are used to estimate the least values of two control parameters that will ensure disease eradication, i.e., the value of *ℛ*_*c*_ to be reduced and kept below unity with baseline values of other parameters fixed. These values of *ξ*_*f*_ and *μ*_*b*_ are estimated at 0.025 and 0.01 so that *ℛ*_*c*_=0.99 or 0.005 and 0.05 with *ℛ*_*c*_=0.95, respectively. Moreover, contour plots of *ℛ*_*c*_ as function of the control strategies with the varying rate of contribution by red foxes to the environment (*η*_*f*_) are computed. The results show remarkable increase in the associated control reproduction number with increase in rate of contribution of parasites to environment by red foxes.

It is worth remarking that amongst the seven most influential parameters globally (*β*_*v*_, *s*, *μ*_*b*_, *η*_*f*_, *ξ*_*f*_, *p*,  *and* *α*_*v*_), five are associated directly with the red foxes, and this justifies our choice of treatment on the population. This also explains the high prevalence of the disease on red foxes as reported in literature.

## Figures and Tables

**Figure 7:**
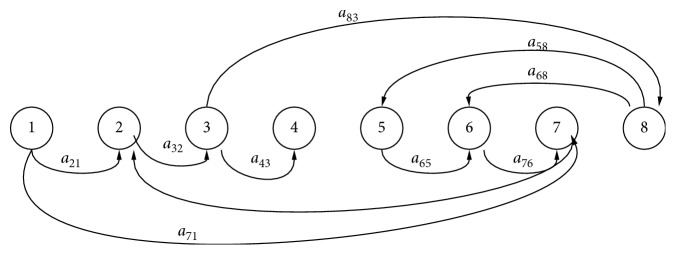
Weighted digraph (*G*, *A*) constructed for models (3)–(10).

**Table 1:** Description of model parameters, indicating baselines, ranges, and references.

Parameter	Description	Baseline value	Value range with time unit 1 day
*μ*_*v*_	Vole per-capita birth rate = death rate	1/(30 × 6)	5 − 7 months [3]
*ξ*_*f*_	Rate of treatment for red fox	0.01	Assumed
*β*_*v*_	Transmission rate from environment to voles	0.004	Assumed
*s*	Searching efficiency of red fox	0.00002	Assumed
*μ*_*f*_	Red fox per-capita birth rate = death rate	1/(365 × 3.5)	2 − 5 years [3]
*p*	Probability that an infected vole preyed on infects a red fox	0.07	[5]
*α*_*v*_	Incubation rate in voles	1/28	25 − 31 days [15]
*α*_*f*_	Incubation rate in red fox	1/75	60 − 90 days [15]
*μ*_*b*_	Rate of cleaning/disinfection of environment	1/31	Assumed
*η*_*f*_	Rate of red fox contribution of *E. multilocularis* to environment	0.02	Assumed
*K*	Concentration of *E. multilocularis* in the feaces that yields 50% chance of catching *Echinococcus*	52	Assumed

**Table 2:** Elasticity indices of *ℛ*_*c*_ relative to model parameters and ranges.

Parameter	Formula ((∂*ℛ*_*c*_/∂*p*)(*p*/*ℛ*_*c*_))	Baseline value	Range	Elasticity index
*β*_*v*_	1/3	0.004	0.00040 − 0.008	0.33333
*s*	−(1/3)	0.00002	(2 × 10^−6^) − (4 × 10^−5^)	−0.33333
*μ*_*b*_	−(1/3)	1/31	(1/62) − (1/3.1)	−0.33333
*η*_*f*_	1/3	0.02	0.002 − 0.04	0.33333
*p*	−(1/3)	0.07	0.007, 0.14	−0.33333
*K*	−(1/3)	52	26 − 78	−0.33333
*ξ*_*f*_	−(1/3)(*ξ*_*f*_/(*μ*_*f*_+*ξ*_*f*_))	0.01	0.001 − 0.02	−0.30913
*α*_*v*_	(1/3)(*μ*_*v*_/(*μ*_*v*_+*α*_*v*_))	1/28	(1/56) − (1/2.8)	0.044872
*α*_*f*_	(1/3)(*μ*_*f*_/(*μ*_*f*_+*α*_*f*_))	1/75	(1/75 × 2) − (1/7.5)	0.018484

## Data Availability

The type of data used in our research are numerical and all the data used are obtained from published literature which are adequately cited therein. Where data are not available (not cited), we use reasonable estimate of the value concern.

## Appendix

### A. Proof of Theorem 1

#### A.1. Existence and Uniqueness of Solutions

Let **X**=(*S*_*f*_, *E*_*f*_, *I*_*f*_, *R*_*f*_, *S*_*v*_, *E*_*v*_, *I*_*v*_, *B*)^*T*^ be a column vector in *ℝ*_+_^8^ so that *x*_1_=*S*_*f*_, *x*_2_=*E*_*f*_, *x*_3_=*I*_*f*_,…, *x*_8_=*B*. Define *f*(**X**)=(*f*_1_(**X**), *f*_2_(**X**),…,*f*_8_(**X**))^*T*^ to be the vector valued function in *ℝ*_+_^8^, where *f*_1_(**X**), *f*_2_(**X**),…, *f*_8_(**X**) are the right-hand sides of model (3)–(10). The model system with initial conditions can therefore be expressed as follows:

(A.1) $$\begin{array}{c} \frac{dX}{dt}=f\left(X\right),\;X\left(0\right)=X_{0},\;\text{where }X:\left[0,\infty \right)⟶{\mathbb{R}}_{+}^{8},f:{\mathbb{R}}_{+}^{8}⟶{\mathbb{R}}_{+}^{8}. \end{array}$$

Therefore, using a standard theorem of the dynamical system [26], *f* is the Lipschitz continuous in **X**. Hence, there exist a unique solution of (3)–(10) for all times *t* > 0.

#### A.2. Positivity of Solutions

Here, we prove that any solution with the nonnegative initial condition that start in *ℝ*_+_^8^ will remain there (i.e., it is positive at all times *t* > 0).

Let (*S*_*f*_(*t*), *E*_*f*_(*t*), *I*_*f*_(*t*), *R*_*f*_(*t*), *S*_*v*_(*t*), *E*_*v*_(*t*), *I*_*v*_(*t*), *B*(*t*)) be any solution of model (3)–(10) with initial conditions (11).

Consider equation (1):

(A.2) $$\begin{array}{c} \frac{dS_{f}}{dt}\geq -\left(spI_{v}+\mu_{f}\right)S_{f}, \end{array}$$

and using the variable separable method, we have

(A.3) $$\begin{array}{c} S_{f}\left(t\right)\geq S_{f}\left(0\right)\text{ exp}\left\{-{\int^{}}_{0}^{t}\left(spI_{v}+\mu_{f}\right)du\right\}. \end{array}$$

From (A.3), it can be seen that

(A.4) $$\begin{array}{c} S_{f}\left(t\right)>0. \end{array}$$

From (7),

(A.5) $$\begin{array}{c} \frac{dS_{v}}{dt}\geq -\left(\beta_{v}\left(\frac{B}{K+B}\right)+\mu_{v}\right)S_{v}, \end{array}$$

so that using the same method as above, we get

(A.6) $$\begin{array}{c} S_{v}\left(t\right)\geq S_{v}\left(0\right)\text{ exp}\left\{-{\int^{}}_{0}^{t}\left(\beta_{v}\left(\frac{B}{K+B}\right)+\mu_{v}\right)du\right\}. \end{array}$$

Hence,

(A.7) $$\begin{array}{c} S_{v}\left(t\right)>0. \end{array}$$

For the positivity of other variables, we proceed by the method of contradiction as follows: Suppose by contradiction that the conclusion is not true, then there exists a time $\bar{t}$ such that

(A.8) $$\begin{array}{c} \text{min}\left\{E_{f}\left(\bar{t}\right),I_{f}\left(\bar{t}\right),R_{f}\left(\bar{t}\right),E_{v}\left(\bar{t}\right),I_{v}\left(\bar{t}\right),B\left(\bar{t}\right)\right\}=0,\\ \text{min}\left\{E_{f}\left(t\right),I_{f}\left(t\right),R_{f}\left(t\right),E_{v}\left(t\right),I_{v}\left(t\right),B\left(t\right)\right\}>0,\text{ for all }t\in \left[0,\;\bar{t}\;\right]. \end{array}$$

Suppose $\text{min}\left\{E_{f}\left(\bar{t}\right),I_{f}\left(\bar{t}\right),R_{f}\left(\bar{t}\right),E_{v}\left(\bar{t}\right),I_{v}\left(\bar{t}\right),B\left(\bar{t}\right)\right\}=E_{f}\left(\bar{t}\right)=0$, then from (4), we have

(A.9) $$\begin{array}{c} \frac{dE_{f}\left(t\right)}{dt}\geq -\left(\mu_{f}+\alpha_{f}\right)E_{f}\left(t\right),\;\text{ for all }t\in \left[0,\bar{t}\;\right], \end{array}$$

so that

(A.10) $$\begin{array}{c} E_{f}\left(\bar{t}\right)\geq E_{f}\left(0\right)\text{ exp}\left(-{\int^{}}_{0}^{\bar{t}}\left(\mu_{f}+\alpha_{f}\right)ds\right)>0, \end{array}$$

which contradicts our earlier assumption. Hence, no such $\bar{t}$ exists. Therefore, *E*_*f*_(*t*) > 0 for all *t* > 0. Suppose also that $\text{min}\left\{E_{f}\left(\bar{t}\right),I_{f}\left(\bar{t}\right),R_{f}\left(\bar{t}\right),E_{v}\left(\bar{t}\right),I_{v}\left(\bar{t}\right),B\left(\bar{t}\right)\right\}=I_{f}\left(\bar{t}\right)=0$, from (5), it implies

(A.11) $$\begin{array}{c} \frac{dI_{f}\left(t\right)}{dt}\geq -\left(\mu_{f}+\xi_{f}\right)I_{f}\left(t\right),\;\text{ for any }t\in \left[0,\bar{t}\;\right]. \end{array}$$

Hence,

(A.12) $$\begin{array}{c} I_{f}\left(\bar{t}\right)>I_{f}\left(0\right)\text{ exp}\left(-{\int^{}}_{0}^{\bar{t}}\left(\mu_{f}+\xi_{f}\right)ds\right)>0, \end{array}$$

which leads to contradiction. Therefore, *I*_*f*_(*t*) > 0 for all *t* > 0.

Similar approaches can be followed to show that *R*_*f*_(*t*) > 0,  *E*_*v*_(*t*) > 0,  *I*_*v*_(*t*) > 0, and *B*(*t*) > 0 for all *t* ∈ (0, *∞*). This completes the proof. Therefore, all solutions of the model (3)–(10) are nonnegative for all *t* > 0.

#### A.3. Boundedness of Solutions

Here, we prove that the solutions are bounded. For the red foxes population, we add equations (3)–(6), and thus,

(A.13) $$\begin{array}{c} \frac{dN_{f}}{dt}=\mu_{f}N_{f}^{*}-\mu_{f}\left(S_{f}+E_{f}+I_{f}+R_{f}\right),\\ =\mu_{f}N_{f}^{*}-\mu_{f}N_{f}^{*}=0. \end{array}$$

Since (*dN*_*f*_/*dt*)=0, this implies that

(A.14) $$\begin{array}{c} N_{f}\left(t\right)=N_{f}^{*}, \end{array}$$

and hence bounded.

From the voles population, also adding equations (7)–(9), we will get

(A.15) $$\begin{array}{c} \frac{dN_{v}}{dt}=\mu_{v}N_{v}^{*}-\mu_{v}\left(S_{v}+E_{v}+I_{v}\right)N_{v},\\ =\mu_{v}N_{v}^{*}-\mu_{v}N_{v}^{*}=0. \end{array}$$

Again, since (*dN*_*v*_/*dt*)=0, this gives

(A.16) $$\begin{array}{c} N_{v}\left(t\right)=N_{v}^{*}. \end{array}$$

and hence bounded above.

Lastly, for the concentration of *Echinococcus*, since *I*_*f*_(*t*) ≤ *N*_*f*_(*t*) ≤ *N*_*f*_^*∗*^, from (10), we have

(A.17) $$\begin{array}{c} \frac{dB}{dt}\leq \eta_{f}N_{f}^{*}-\mu_{b}B. \end{array}$$

Hence,

(A.18) $$\begin{array}{c} B\left(t\right)\leq \frac{\eta_{f}N_{f}^{*}}{\mu_{b}}. \end{array}$$

### B. Calculation of Control Reproduction Number

Using the approach in [19], we define the matrix *F* as the generation of secondary infectious cases and *V* as the matrix of transition rates between the infected compartments. The infected compartments are listed in order for easy application of the method as (*E*_*f*_, *I*_*f*_, *R*_*f*_, *E*_*v*_, *I*_*v*_, *B*). Hence, *F* and *V* are calculated as follows:

(B.1) $$\begin{array}{c} F=\left[\begin{array}{cccccc} 0 & 0 & 0 & 0 & spN_{f}^{*} & 0\\ 0 & 0 & 0 & 0 & 0 & 0\\ 0 & 0 & 0 & 0 & 0 & 0\\ 0 & 0 & 0 & 0 & 0 & \frac{\beta_{v}N_{v}^{*}}{K}\\ 0 & 0 & 0 & 0 & 0 & 0\\ 0 & \eta_{f} & 0 & 0 & 0 & 0 \end{array}\right],\\ V=\left[\begin{array}{cccccc} {\bar{\alpha }}_{f} & 0 & 0 & 0 & 0 & 0\\ -\alpha_{f} & {\bar{\xi }}_{f} & 0 & 0 & 0 & 0\\ 0 & \xi_{f} & \mu_{f} & 0 & 0 & 0\\ 0 & 0 & 0 & {\bar{\alpha }}_{v} & 0 & 0\\ 0 & 0 & 0 & -\alpha_{v} & \mu_{v} & 0\\ 0 & 0 & 0 & 0 & 0 & \mu_{b} \end{array}\right], \end{array}$$

where ${\bar{\alpha }}_{f}=\mu_{f}+\alpha_{f},\;{\bar{\xi }}_{f}=\mu_{f}+\xi_{f}\text{ and }{\bar{\alpha }}_{v}=\alpha_{v}+\mu_{v}$.

The inverse of *V* is obtained as follows:

(B.2) $$\begin{array}{c} V^{-1}=\left[\begin{array}{cccccc} \frac{1}{{\bar{\alpha }}_{f}} & 0 & 0 & 0 & 0 & 0\\ \frac{\alpha_{f}}{{\bar{\alpha }}_{f}{\bar{\xi }}_{f}} & \frac{1}{{\bar{\xi }}_{f}} & 0 & 0 & 0 & 0\\ \frac{\alpha_{f}\xi_{f}}{{\bar{\alpha }}_{f}{\bar{\xi }}_{f}\mu_{f}} & \frac{1}{\mu_{f}} & 0 & 0 & 0 & \\ 0 & 0 & 0 & \frac{1}{{\bar{\alpha }}_{v}} & 0 & 0\\ 0 & 0 & 0 & \frac{\alpha_{v}}{\mu_{v}{\bar{\alpha }}_{v}} & \frac{1}{\mu_{v}} & 0\\ 0 & 0 & 0 & 0 & 0 & \frac{1}{\mu_{b}} \end{array}\right], \end{array}$$

and hence,

(B.3) $$\begin{array}{c} FV^{-1}=\left[\begin{array}{cccccc} 0 & 0 & 0 & \frac{spN_{f}^{*}\alpha_{v}}{{\bar{\alpha }}_{v}\mu_{v}} & \frac{spN_{f}^{*}}{\mu_{v}} & 0\\ 0 & 0 & 0 & 0 & 0 & 0\\ 0 & 0 & 0 & 0 & 0 & 0\\ 0 & 0 & 0 & 0 & 0 & \frac{\beta_{v}N_{v}^{*}}{\mu_{b}K}\\ 0 & 0 & 0 & 0 & 0 & 0\\ \frac{\eta_{f}\alpha_{f}}{{\bar{\alpha }}_{f}{\bar{\xi }}_{f}} & \frac{\eta_{f}}{{\bar{\xi }}_{f}} & 0 & 0 & 0 & 0 \end{array}\right]. \end{array}$$

Therefore, the basic control reproduction number, denoted by *ℛ*_*c*_, is obtained by taking the spectral radius of the *FV*^−1^ matrix, and thus,

(B.4) $$\begin{array}{c} {ℛ}_{c}=\rho \left(FV^{-1}\right)={\left[\left(\frac{N_{f}^{*}sp\alpha_{f}}{{\bar{\alpha }}_{f}{\bar{\xi }}_{f}}\right)\left(\frac{N_{v}^{*}\beta_{v}\alpha_{v}}{\mu_{v}{\bar{\alpha }}_{v}}\right)\left(\frac{\eta_{f}}{K\mu_{b}}\right)\right]}^{1/3}. \end{array}$$

### C. Proof of Theorem 4

Proof At equilibrium, equations of system (1) can be expressed as follows:

(C.1) $$\begin{array}{c} \mu_{f}N_{f}^{*}=spI_{v}^{*}S_{f}^{*}+\mu_{f}S_{f}^{*}, \end{array}$$

(C.2) $$\begin{array}{c} {\bar{\alpha }}_{f}=\frac{spI_{v}^{*}S_{f}^{*}}{E_{f}^{*}}, \end{array}$$

(C.3) $$\begin{array}{c} {\bar{\xi }}_{f}=\frac{\alpha_{f}E_{f}^{*}}{I_{f}^{*}}, \end{array}$$

(C.4) $$\begin{array}{c} \mu_{f}=\frac{\xi_{f}I_{f}^{*}}{R_{f}^{*}}, \end{array}$$

(C.5) $$\begin{array}{c} \mu_{v}N_{v}^{*}=\beta_{v}\frac{B^{*}}{\kappa +B^{*}}S_{v}^{*}+\mu_{v}S_{v}^{*}, \end{array}$$

(C.6) $$\begin{array}{c} {\bar{\alpha }}_{v}=\beta_{v}\frac{B^{*}}{\kappa +B^{*}}\frac{S_{v}^{*}}{E_{v}^{*}}, \end{array}$$

(C.7) $$\begin{array}{c} \mu_{v}=\frac{\alpha_{v}E_{v}^{*}}{I_{v}^{*}}, \end{array}$$

(C.8) $$\begin{array}{c} \mu_{b}=\frac{\eta_{f}I_{f}^{*}}{B^{*}}. \end{array}$$

Let

(C.9) $$\begin{array}{c} L_{1}=S_{f}-S_{f}^{*}-S_{f}^{*}\text{ ln}\frac{S_{f}}{S_{f}^{*}},\\ L_{2}=E_{f}-E_{f}^{*}-E_{f}^{*}\text{ ln}\frac{E_{f}}{E_{f}^{*}},\\ L_{3}=I_{f}-I_{f}^{*}-I_{f}^{*}\text{ ln}\frac{I_{f}}{I_{f}^{*}},\\ L_{4}=R_{f}-R_{f}^{*}-R_{f}^{*}\text{ ln}\frac{R_{f}}{R_{f}^{*}},\\ L_{5}=S_{v}-S_{v}^{*}-S_{v}^{*}\text{ ln}\frac{S_{v}}{S_{v}^{*}},\\ L_{6}=E_{v}-E_{v}^{*}-E_{v}^{*}\text{ ln}\frac{E_{v}}{E_{v}^{*}},\\ L_{7}=I_{v}-I_{v}^{*}-I_{v}^{*}\text{ ln}\frac{I_{v}}{I_{v}^{*}},\\ L_{8}=B-B^{*}-B^{*}\text{ ln}\frac{B}{B^{*}}. \end{array}$$

Differentiating *L*_*i*_ for *i*=1,2,…, 8 along solutions of system (3)–(10) and using the inequality 1 − *y*+*lny* ≤ 0 for *y* > 0 with equality only if *y*=0, we have

(C.10) L1′=1−Sf∗SfSf′=1−Sf∗SfμfNf∗−spIvSf−μfSf=1−Sf∗SfspIv∗Sf∗+μfSf∗−spIvSf−μfSf, from C.1=spIv∗Sf∗1−Sf∗Sf1−IvSfIv∗Sf∗+μfSf∗1−Sf∗Sf1−SfSf∗=spIv∗Sf∗1−IvSfIv∗Sf∗−Sf∗Sf+IvIv∗+μfSf∗1−SfSf∗−Sf∗Sf+1=spIv∗Sf∗lnSf∗Sf−Sf∗Sf+IvIv∗−lnIvIv∗+μfSf∗2−SfSf∗−Sf∗Sf≤spIv∗Sf∗lnSf∗Sf−Sf∗Sf+IvIv∗−lnIvIv∗:=a71G71,  where  a71=spIv∗Sf∗,L2′=1−Ef∗EfEf′=1−Ef∗EfspIvSf−α¯fEf=1−Ef∗EfspIvSf−spIv∗Sf∗EfEf∗, from C.2=spIv∗Sf∗IvSfIv∗Sf∗−EfEf∗−IvSfEf∗Iv∗Sf∗Ef+1≤spIv∗Sf∗IvSfIv∗Sf∗−lnIvSfIv∗Sf∗−EfEf∗+lnEfEf∗≤spIv∗Sf∗IvSfIv∗Sf∗−lnIvSfIv∗Sf∗+spIv∗Sf∗−EfEf∗+lnEfEf∗:=a21G21+a27G27,  where  a21=a27=spIv∗Sf∗,L3′=1−If∗IfIf′=1−If∗IfαfEf−ξf¯If=1−If∗IfαfEf−αfEf∗IfIf∗, from C.3=αfEf−αfEf∗IfIf∗−αfEfIf∗If+αfEf∗=αfEf∗EfEf∗−IfIf∗−If∗EfIfEf∗+1=αfEf∗EfEf∗−lnEfEf∗−IfIf∗+lnIfIf∗:=a32G32,  where  a32=αfEf∗,L4′=1−Rf∗RfRf′=1−Rf∗RfξfIf−μfRf=1−Rf∗RfξfIf−ξfIf∗RfRf∗,from C.4=ξfIf−ξfIf∗RfRf∗−ξfIfRf∗Rf+ξfIf∗=ξfIf∗IfIf∗−RfRf∗−Rf∗IfRfIf∗+1≤ξfIf∗IfIf∗−lnIfIf∗−RfRf∗+lnRfRf∗:=a43G43,   where a43=ξfIf∗,L5′=1−Sv∗SVSv′=1−Sv∗SVμvNv∗−βvBK+BSv−μvSv=1−Sv∗SvβvB∗K+B∗Sv∗+μvSv∗−βvBK+BSv−μvSv, from C.5=1−Sv∗SvβvB∗K+B∗Sv∗−βvBK+BSv+1−Sv∗SvμvSv∗−μvSv=βvB∗Sv∗K+B∗1−Sv∗Sv1−BSvK+BB∗Sv∗K+B∗+μvSv∗1−Sv∗Sv1−SvSv∗=βvB∗Sv∗K+B∗1−BSvB∗Sv∗K+BK+B∗−Sv∗Sv+BK+B∗B∗K+B+μvSv∗1−SvSv∗−Sv∗Sv+1≤βvB∗Sv∗K+B∗BK+B∗B∗K+B−lnBK+B∗B∗K+B−Sv∗Sv+lnSv∗Sv:=a58G58,  where  a58=βvB∗Sv∗K+B∗,L6′=1−Ev∗EVEv′=βvBK+BSv−α¯vEv=1−Ev∗EvβvBSvK+B−βvB∗Sv∗EvK+B∗Ev∗, from C.6=βvBSvK+B−βvB∗Sv∗EvK+B∗Ev∗−βvBSvEv∗K+BEv+βvB∗Sv∗K+B∗=βvB∗Sv∗K+B∗BSvK+B∗K+BB∗Sv∗−EvEv∗−BSvEv∗K+B∗B∗Sv∗EvK+B+1≤βvB∗Sv∗K+B∗BSvK+B∗B∗Sv∗K+B−lnBSvK+B∗B∗Sv∗K+B−EvEv∗+lnEvEv∗:=a68G68+a65G65,   where a65=a68=βvB∗Sv∗K+B∗,L7′=1−Iv∗IvIv′=1−Iv∗IvαvEv−μvIv=1−Iv∗IvαvEv−αvEv∗IvIv∗, from C.7=αvEv−αvEv∗IvIv∗−αvEvIv∗Iv+αvEv∗=αvEv∗EvEv∗+IvIv∗−Iv∗EvIvEv∗+1≤αvEv∗EvEv∗−lnEvEv∗−IvIv∗+lnIvIv∗:=a76G76,  where  a76=αvEv∗,L8′=1−B∗BB′=1−B∗BηfIf−μbB=1−B∗BηfIf−ηfIf∗BB∗, from C.8=ηfIf−ηfIf∗BB∗−ηfIfB∗B+ηfIf∗=ηfIf∗IfIf∗−BB∗−IfB∗If∗B+1≤ηfIf∗IfIf∗−lnIfIf∗−BB∗+lnBB∗:=a83G83,   where a83=ηfIf∗.

From the derivatives of *L*_*i*_ for *i*=1,2,3,…, 8, it can be seen that all *a*_*i*_ ≥ 0 and the digraph does not have any cycle, as shown in Figure 7. Using the application of Theorems 3.3 and 3.5 of [20], ∃*c*_*i*_, 1 ≤ *i* ≤ 8 so that *L*=∑_*i*=1_^8^*c*_*i*_*L*_*i*_ gives a Lyapunov function for system (3)–(10), where *c*_*i*_'s can be determined as follows: Since *d*^−^(5)=1, *c*_5_*a*_58_=*c*_6_*a*_65_⟹*c*_6_=(*a*_65_/*a*_58_)*c*_5_. Given *d*^+^(6)=1, *c*_7_*a*_76_=*c*_6_*a*_65_+*c*_6_*a*_68_⟹*c*_7_=((*a*_65_(*a*_65_+*a*_68_))/*a*_58_*a*_76_). From *d*^+^(1)=1, *c*_7_*a*_17_=*c*_2_*a*_27_⟹*c*_2_=(*a*_17_/*a*_21_)*c*_7_=((*a*_65_*a*_71_(*a*_65_+*a*_68_))/*a*_21_*a*_58_*a*_76_). From *d*^−^(7)=1, *c*_7_*a*_76_=*c*_1_*a*_17_+*c*_2_*a*_27_=*c*_1_*a*_17_+*c*_1_*a*_17_=2*c*_1_*a*_17_

(C.11) $$\begin{array}{c} ⟹c_{1}=\frac{a_{76}}{2a_{17}}c_{7}. \end{array}$$

Therefore,

(C.12) $$\begin{array}{c} c_{2}=\frac{a_{76}a_{65}\left(a_{65}+a_{68}\right)}{a_{58}a_{76}}c_{5}. \end{array}$$

From *d*^+^(2)=1,

(C.13) $$\begin{array}{c} c_{2}a_{27}+c_{2}a_{21}=c_{3}a_{32}⟹c_{3}=\frac{\left(a_{27}+a_{21}\right)}{a_{32}}=\frac{a_{76}a_{65}\left(a_{65}+a_{68}\right)}{a_{58}a_{76}}c_{5}\frac{\left(a_{27}+a_{21}\right)}{a_{32}}. \end{array}$$

And when *d*^−^(8)=1,

(C.14) $$\begin{array}{c} c_{8}a_{83}=c_{5}a_{58}+c_{6}a_{68}⟹c_{8}=\left(\frac{a_{58}+\left(a_{65}/a_{58}\right)}{a_{83}}\right)c_{5}. \end{array}$$

It can easily be verified that {*ℰ*^*∗*^} is the only invariant set in the interior of *Ω* that can satisfy *L*′=0 and thus the uniqueness of *ℰ*^*∗*^. Using this Lyapunov function in combination with LaSalle's invariance principle [21] completes the proof that *ℰ*^*∗*^ is unique and globally asymptotically stable in *Ω* if *ℛ*_*c*_ > 1.

## References

[1] Eckert Z., Gemmel J., Meslin M. A., Pawlowski F.-X. (2001). *WHO/OIE Manual on Echinococcosis in Humans and Animals: A Public Health Problem of Global Concern*.

[2] Mastin A. J. (2015). *Canine Echinococcosis in Kyrgyzstan: Detection, Diagnosis, and Dynamics*.

[3] Roberts M. G., Aubert M. F. A. (1995). A model for the control of Echinococcus multilocularis in France. *Veterinary Parasitology*.

[4] Eckert J., Deplazes P. (2004). Biological, epidemiological, and clinical aspects of echinococcosis, a zoonosis of increasing concern. *Clinical Microbiology Reviews*.

[5] Doi R., Ishikawa H., Ohga Y. (2003). A model for the transmission of Echinococcus multilocularis in Hokkaido, Japan. *Parasitology Research*.

[6] Budke C. M., Jiamin Q., Craig P. S., Torgerson P. R. (2005). Modeling the transmission of Echinococcus granulosus and Echinococcus multilocularis in dogs for a high endemic region of the Tibetan plateau. *International Journal for Parasitology*.

[7] Lewis F. I., Otero-Abad B., Hegglin D., Deplazes P., Torgerson P. R. (2014). Dynamics of the force of infection: insights from echinococcus multilocularis infection in foxes. *PLoS Neglected Tropical Diseases*.

[8] Craig P. S., Li T., Qiu J. (2008). Echinococcoses and Tibetan communities. *Emerging Infectious Diseases*.

[9] Davidson R. K., Romig T., Jenkins E., Tryland M., Robertson L. J. (2012). The impact of globalisation on the distribution of Echinococcus multilocularis. *Trends in Parasitology*.

[10] Maudlin I., Eisler M. C., Welburn S. C. (2009). Neglected and endemic zoonoses. *Philosophical Transactions of the Royal Society B: Biological Sciences*.

[11] Molyneux D., Hallaj Z., Keusch G. T. (2011). Zoonoses and marginalised infectious diseases of poverty: where do we stand?. *Parasites and Vectors*.

[12] Umhang G., Karamon J., Hormaz V., Knapp J., Cencek T., Boué F. (2017). A step forward in the understanding of the presence and expansion of Echinococcus multilocularis in Eastern Europe using microsatellite EmsB genotyping in Poland. *Infection, Genetics and Evolution*.

[13] Hofer S., Gloor S., Müller U., Mathis A., Hegglin D., Deplazes P. (2000). High prevalence of Echinococcus multilocularis in urban red foxes (*Vulpes vulpes*) and voles (*Arvicola terrestris*) in the city of Zürich, Switzerland. *Parasitology*.

[14] Karamon J., Kochanowski M., Sroka J. (2014). The prevalence of Echinococcus multilocularis in red foxes in Poland-current results (2009-2013). *Parasitology Research*.

[15] Miller A. L. (2016). *The Role of Rodents in the Transmission of Echinococcus Multilocularis and Other Tapeworms in a Low Endemic Area*.

[16] Otero-Abad B., Rüegg S. R., Hegglin D., Deplazes P., Torgerson P. R. (2017). Mathematical modelling of Echinococcus multilocularis abundance in foxes in Zurich, Switzerland. *Parasites and Vectors*.

[17] Wang Y., Wei J. (2012). Global dynamics of a cholera model with time delay. *International Journal of Biomathematics*.

[18] Rong X., Fan M., Sun X., Wang Y., Zhu H. (2018). Impact of disposing stray dogs on risk assessment and control of Echinococcosis in Inner Mongolia. *Mathematical Biosciences*.

[19] van den Driessche P., Watmough J. (2002). Reproduction numbers and sub-threshold endemic equilibria for compartmental models of disease transmission. *Mathematical Biosciences*.

[20] Shuai Z., van den Driessche P. (2013). Global stability of infectious disease models using Lyapunov functions. *SIAM Journal on Applied Mathematics*.

[21] LaSalle J. P. (1976). *The Stability of Dynamical Systems, Regional Conference Series in Applied Mathematics*.

[22] Chitnis N., Hyman J. M., Cushing J. M. (2008). Determining important parameters in the spread of malaria through the sensitivity analysis of a mathematical model. *Bulletin of Mathematical Biology*.

[23] Martcheva M. (2015). *An Introduction to Mathematical Epidemiology*.

[24] Bürli C., Harbrecht H., Odermatt P., Sayasone S., Chitnis N. (2018). Mathematical analysis of the transmission dynamics of the liver fluke, Opisthorchis viverrini. *Journal of Theoretical Biology*.

[25] Marino S., Hogue I. B., Ray C. J., Kirschner D. E. (2008). A methodology for performing global uncertainty and sensitivity analysis in systems biology. *Journal of Theoretical Biology*.

[26] Stuart A., Humphries A. R. (1998). *Dynamical Systems and Numerical Analysis*.

